# Prevention of Migrating Atrial Septal Occluder in Large Hole Atrial Septal Defect Intervention by Snare

**DOI:** 10.7759/cureus.36904

**Published:** 2023-03-30

**Authors:** Lam Truong Hoai, Duc Nguyen Hung, Kien Nguyen Trung, Duyen Nguyen Thi

**Affiliations:** 1 Cardiology, Tâm Anh Hospital, Ha Noi, VNM; 2 Cardiology, Tam Anh Hospital, Ha Noi, VNM; 3 Cardiology, Bach Mai Hospital, Ha Noi, VNM

**Keywords:** snare., embolization, device retrieval, deficient margin, amplatzer device, atrial septal defect

## Abstract

Migrating Amplatzer Septal Occluder (ASO) is a rare complication due to insufficient margins, especially large-hole Atrial Septal Defect (ASD). After deploying, ASO occasionally exposes the low margins, resulting in dislocated devices and embolization. The majority of embolizations happen right away after release. The embolized device must be removed using extended fluoroscopy and occasionally by open heart surgery. The device is released by unscrewing the cable while the snare holds the screw end. On Transesophageal Echocardiography (TEE), the device position is once again validated. If the device is stable, the snare is then removed.

## Introduction

One of the most prevalent lesions associated with adult congenital heart disease is the atrial septal Secundum defect. For acceptable secundum Atrial Septal Defect (ASD), transcatheter ASD closure is now a commonly accepted substitute for surgical closure. Not all ASDs, however, have anatomical conditions that allow for transcatheter closure. To position the ASD device, the size of the ASD should be kept to a minimum, and there should be enough interatrial septal tissue around the defect to protect surrounding structures. Recognition of morphological changes in secundum-type ASD is essential for determining whether patients are candidates for percutaneous closure [1]. For imaging in ASD closure, transesophageal echocardiography (TEE) continues to be the gold standard. The evaluation of ASD using TEE comprises determining the number and location of the defect(s), as well as the size and sufficiency of the rims [2]. Following device closures, a few problems have been documented; however, they are rare. The most frequent justification for surgical intervention is malposition or embolization. Ten patients required surgical intervention out of the 124 who underwent percutaneous closure of ASD [3]. Due to device malposition (in three patients) or device embolization, ten patients had elective surgical repair (seven patients) of the 417 patients described by Chessa et al. [4] who required surgical intervention due to embolization or malposition. Within minutes of unscrewing the device delivery cable, patients with big defects and inadequate margins tend to undergo the majority of embolizations. As long as the device is still connected to the rigid delivery wire, it may appear to be in a stable position even though it is not being held securely by inadequate margins. The rigid cable only partially supports the device; it is held securely in certain margins but could be insecure and unstable in others. We can identify unstable device locations and devices on the verge of embolization if there is a mechanism to hold the device even after unscrewing the rigid delivery cable wire [5].

We propose using a snare to hold the device preventatively before releasing it from the delivery cable to make it easier to retrieve if it is displaced before embolization.

## Case presentation

We described the closure of a significant ostium Secundum ASD, measured at 31 mm on transesophageal echo (TEE), and was closed with 42 mm due to insufficient rims (Lifetech Scientific Inc., China). 

**Figure 1:**
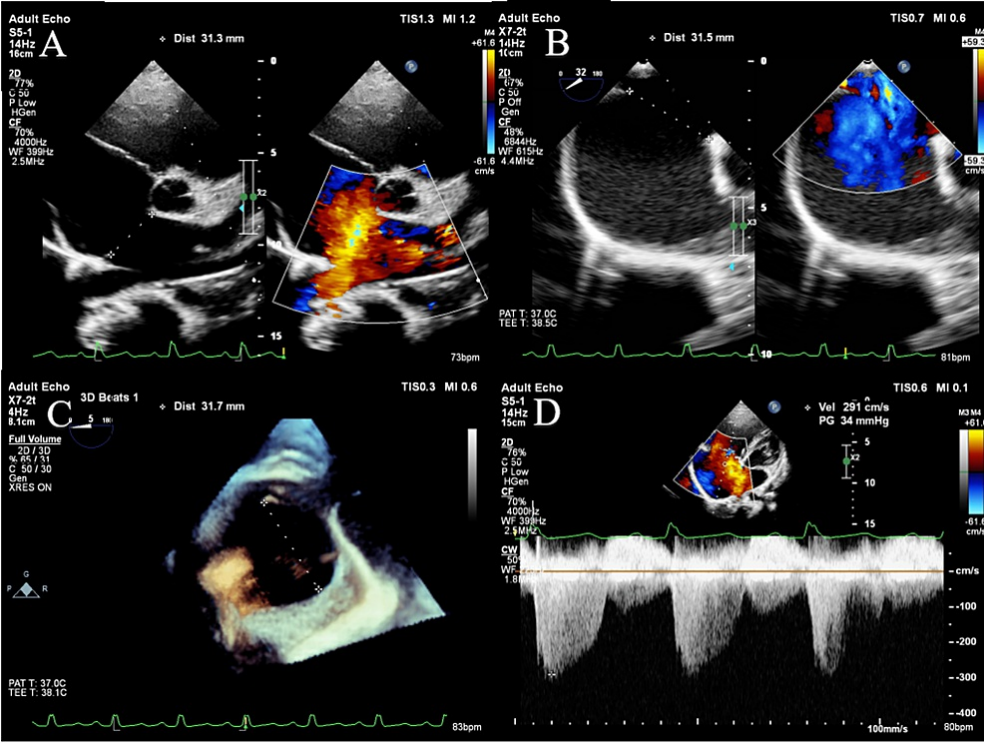
1A: ASD size on TTE; 1B: ASD size on TEE ; 1C: ASD size on 3D TEE; 1D Pulmonary hypertension ASD: Atrial Septal Defect; TEE: Transesophageal Echocardiography

A 16-year-old female complained of palpitations and shortness of breath with exertion. Upon examination, her second heart sound was wide and fixed, and there was a mild ejection systolic murmur across her left second and third intercostal spaces. A large ASD measuring 31.3 mm was found on the TTE (Figure 1A) and 31.5 mm on the TEE (Figure 1B), along with evidence of mild pulmonary arterial hypertension (figure 1D), Right ventricle was mildly dilated, the biventricular function was normal, and insufficient septal tissue rims all around (Inferior vena cava rim was 3.6mm and the aortic rim was 4.2mm). The patient wanted to avoid surgery because she was a teenager, so an ASD device closure trial was chosen as the therapy method.

**Figure 2:**
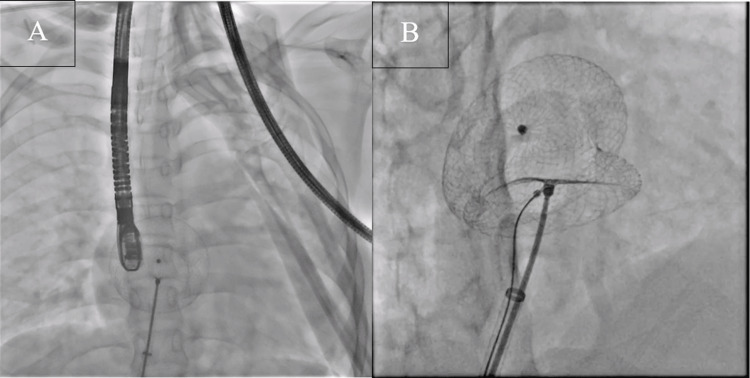
2A: TEE guidance intervention; 2B To grab the device's screw end and unscrew it, a snare is passed alongside the delivery cable.

The 7 French venous sheaths were exchanged for 14 French device delivery sheaths. The device was first placed in the left upper pulmonary vein (LUPV) with part of the left atrial disc in the LUPV but failed. Hence we inserted a portion of the left atrial disc into the right upper pulmonary vein (RUPV) along with the device on the second try. Then the right atrial disc was delivered to the other side, followed by the left one. The left atrial disc slid into the corresponding position and completely covered the shunt after confirming that the device was in the correct position. The TEE recorded the absence of residual shunt. The snare slid under the cable in the delivery to the crew of the right atrial disc (figure 2). The loop on the right atrial disc screw was tightened once the snare arrived at the right atrial screw end of the cable. The delivery cable was given a light Minnesota wiggle to check the stability of the ASO. The snare was still attached to the device's screw end as the delivery cable was unscrewed (Video 1). The delivery cable was released from the screw end, and the device then realigned itself along the interatrial septal plane's natural lay. The device's stability and absence of residual flows were established on echocardiography. The snare was subsequently removed.

**Video 1:** The snare was still attached to the device's screw end as the delivery cable was then unscrewed

## Discussion

In the modern era, device closure is the therapy for secundum ASD, and short recuperation time is one of its main benefits. It is common knowledge that ASDs exhibit morphological differences, making the proper patient selection for transcatheter ASD closure essential to a successful operation. In this case, the aortic rim was insufficient, but this is often as was in the series of patients who showed 85% absents [6]. The new prevalence of 30 to 50% has the defective aortic rim, which absence increases procedural complexity in device closure ASDs. More than 40% of patients with ASD have an aortic rim less than 5 mm; therefore, aortic rim deficit will be widespread if this is the accepted standard (1). ASO impact on the aorta is more likely when the aortic rim is insufficient. Aortic erosion, a rare but possibly fatal consequence, has sparked discussion over the safety of occluding ASD using a device. Deficient retro-aortic rim and device-to-defect oversizing were identified in a review of documented cases of aortic erosion as potential risk factors.

The IVC rim is crucial for proper ASD device closure. Device closure of an ASD is deemed contraindicated in the absence of an IVC rim. Transcatheter closure of this form of ASD is complex and frequently accompanied by problems such as residual shunts, future malposition, and embolization of the device due to insufficient IVC rim. Transesophageal echocardiography (TEE), as opposed to traditional TTE, provides a better assessment of the flaws' size, number, and position in addition to insufficient IVC rim. The other rims should be adequate. We used TEE initially to acquire experience before converting it to a simpler TTE. TTE is a trustworthy method for measuring ASD diameters and circumferential margins. The distance between the margin of the ASD and the superior vena cava, right pulmonary vein, aortic root, atrioventricular valves, and coronary sinus can be fairly estimated by TTE, which is especially true of the IVC rim. It has also helped observe and direct the placement of devices.

The rigid delivery cable may retain the precariously implanted device over the atrial septum without any considerable or discernible residual flow during the deployment of a device across a large ASD with insufficient rims. Our patients' tiny inferior rim and posterior margin deficiency may have contributed to device malposition. We purposely selected a large device. When the delivery cable was released, a snare attached to the right atrial disc's screw helped prevent embolization of the misplaced device and made device retrieval quite simple and quick. With the snare's ability to control the device, the percutaneous recovery of big devices was easier. Large embolized Amplatzer device retrieval by transcatheter was once considered challenging, and surgical retrieval techniques were frequently employed [7]. The delivery cable's stiff support for the device is withdrawn when it has been unscrewed, and the margins it holds are then again evaluated on echocardiography. The soft snare catheter is not supported by the device, which is still gripping the screw. Devices are retrieved with snares if device malposition or residual flow across any margin is discovered. In recovery, holding the screw with the snare also makes it easier to align the device with the lengthy sheath.

## Conclusions

We suggest using this proper technique, particularly when deploying larger devices in ASD with inadequate rims to prevent closing a large ASD with a defective rim can be difficult, and device misposition and embolization can make matters worse. This snare-assisted device release method prevents device embolization in large ASDs with insufficient rims and makes retrieval easier.
